# Healing rates in diabetes-related foot ulcers using low frequency ultrasonic debridement versus non-surgical sharps debridement: a randomised controlled trial

**DOI:** 10.1186/s13104-018-3841-4

**Published:** 2018-10-16

**Authors:** Lucia Michailidis, Shan M. Bergin, Terry P. Haines, Cylie M. Williams

**Affiliations:** 1 Podiatry Department, Monash Health, Monash Medical Centre, 246 Clayton Road, Clayton, VIC 3168 Australia; 2 Physiotherapy Department, School of Primary and Allied Health Care, Monash University, McMahons Rd, Frankston, VIC 3199 Australia; 3 Peninsula Health, Allied Health, 4 Hastings Rd, Frankston, VIC 3199 Australia

**Keywords:** Debridement, Diabetes complications, Wound healing, Ultrasonics

## Abstract

**Objective:**

Current clinical practice varies around debridement techniques used to promote healing of diabetes-related foot ulcers. This randomised controlled study will compare healing rates for diabetes-related foot ulcers treated with low frequency ultrasonic debridement versus non-surgical sharps debridement. Individuals with diabetes-related foot ulcers being managed by podiatry at a metropolitan hospital were screened against study criteria. Eligible participants were randomly allocated to either the non-surgical sharps debridement group or the low frequency ultrasonic debridement group and received weekly treatment for 6 months. Participants also completed a quality of life measure and visual analogue pain scale.

**Results:**

This trial was ended early due to recruitment issues. Ten participants with 14 ulcers participated. Results were analysed using a survival analysis approach. Ulcers treated with non-surgical sharps debridement healed more quickly (61.6 days ± 24.4) compared with low frequency ultrasonic debridement (117.6 days ± 40.3). In both groups, quality of life was observed to improve as ulcers healed and pain levels reduced as ulcers improved. Observations from this study found faster healing using non-surgical sharps debridement. However, these results are unable to be generalised due to the small sample size. Further research is recommended.

*Trial registration* Australian New Zealand Clinical Trial Registry: ACTRN12612000490875

## Introduction

Diabetes and its complications are rapidly becoming the world’s most significant cause of morbidity and mortality. Globally, the number of adults with diagnosed diabetes is approximately 415 million [1] or one in eleven adults, a worldwide prevalence that was previously predicted to occur in 2030 [2].

Diabetic foot disease is also considered one of the most serious complications of diabetes. The pathophysiology is multifactorial and is predominantly associated with neuropathy, peripheral arterial disease and foot deformity [3–6]. The convergence of one or more of these conditions leads to the development of foot ulceration, which is a significant precursor to lower limb amputation [7]. It is estimated that up to 25% of people with diabetes will develop a foot ulcer in their lifetime, making them 36 times more likely to experience subsequent amputation [7, 8].

The treatment goal for diabetes-related foot ulcers (DRFUs) is to achieve healing as quickly as possible to prevent the onset of serious complications. Treatment commonly includes antibiotic therapy for infection, re-vascularisation in the presence of reduced arterial perfusion, offloading of pressure, appropriate dressings and regular debridement of non-viable tissue [6, 7]. Debridement is fundamental in DRFU management [6] and facilitates healing by ensuring the best possible preparation of the wound bed and margins [9, 10]. Many different methods of debridement exist but there is very little evidence to support a single method or the frequency that it should be performed [6, 10]. Similarly, there are variable costs of debridement methods and there is little economic evaluation of cost versus effectiveness to guide clinicians to make economically feasible treatment choices [11].

The primary outcome of this study is proportion of DRFUs healed using non-surgical sharps debridement (NSSD) versus low frequency ultrasonic debridement (LFUD) over a 6-month period. Secondary outcomes include quality of life measure and assessment of pain before, during and after treatment. This study adhered to a previously published protocol [12].

## Main text

### Participants and setting

From March 2013 to February 2015 all patients with a DRFU receiving treatment by podiatry at Monash Health, Victoria, Australia, were screened against the study inclusion criteria (Table 1).

**Table 1:** Participant inclusion and exclusion criteria

Inclusion criteria	Exclusion criteria
General: ≥ 30 years of age Able to provide informed consent Ulcers present for ≥ 1 month Ulcers ≥ 1 cm^2^	General: Patients taking immunosuppressive medications Known allergy to ulcer dressing products Pre-existing ulcer pain preventing either type of debridement
Vascular: Palpable pedal pulses OR toe pressure ≥ 45 mmHg OR those meeting Rutherford Classification of peripheral arterial disease stages [13]: 0 (Asymptomatic) 1 (Mild claudication) 2 (Moderation claudication at 200 m)	Vascular: Those meeting Rutherford Classification of peripheral arterial disease stages: 3 (Severe claudication) 4 (Rest pain) 5 (Ischaemic ulceration no exceeding ulcer of the digits of the toes) 6 (Severe ischaemic ulcers of frank gangrene)
Ulcer classification: Infected ulcers being appropriately managed Those meeting The University of Texas Wound classification criteria [14]: A1, A2, A3 (wounds of varying depth without infection or ischaemia) B1, B2, B3 (wounds of varying depth with infection only)	Wound classification: Dry gangrenous ulcer Fungating ulcers Malignant ulcers Those meeting the University of Texas wound classification criteria: A0, B0, C0, D0 (pre or post-ulcerative lesion with complete epithelialisation, with or without infection and ischaemia) C1, C2, C3 (wounds of varying depth with ischaemia only) D1, D2, D3 (wound of varying depth with infection and ischaemia

Participants identified as meeting the study criteria were informed about the research project by the treating podiatrist. Those agreeable to participating were provided with a patient information and consent form and written consent was obtained. Approval was granted by the Monash Health Human Research Ethics Committee (HREC Reference Number 12101B).

### Interventions

The two interventions included LFUD (intervention) and NSSD (control), which were applied according to a standardised technique. Debridement occurred weekly until healing occurred. The time of each debridement was performed for as long as required to remove as much non-viable tissue as possible from the wound base. Wound dressings, pressure offloading and footwear were applied according to evidence-based practice [6]. This was decided by the treating podiatrist based on clinical need, ulcer appearance and location.

Participant quality of life was assessed at baseline, 3 months and at 6 months or once healed using the validated tool EQ-5D-5L [15]. Where multiple ulcers existed at the same time, on a single participant, and resolved at the same time, the data was represented only once. Ulcer pain was measured before, during and after each debridement using a validated 100 mm Visual Analogue Scale [16].

### Outcomes

The primary outcome measure for this study was the proportion of DRFUs healed over the 6-month follow up period. Healing was determined by assessing the total surface area of the ulceration site. Ulcer depth was measured by the treating podiatrist using a disposable probe at the deepest point following each debridement. Where the ulcer depth could not be measured (less than 0.1 cm) but the ulcer remained unhealed, a standardised depth of 0.1 cm was used. Ulcer undermining was also measured following each debridement using a disposable probe. The extent of undermining was marked on the skin with a black marker.

Photographs were taken using a digital camera following each debridement. A standardised technique was implemented to reduce variation in photographic angles. Calculation of wound surface area was undertaken at the completion of the study by a member of the research team not involved in data collection (CW). A previously established inter-rater measurement reliability of calculating wound surface area between the treating podiatrist and a research team member was 0.99.

Secondary outcome measures included ulcer pain before, during and after each debridement using a validated 100 mm Visual Analogue Scale (VAS) [16]. Health related quality of life was assessed using the validated EQ-5D-5L [15]. This tool analyses five health-related quality of life domains including mobility, self-care, usual activities, pain/discomfort and anxiety/depression. Participants completed this at the initial treatment, 3 months and again at 6 months or the final appointment.

### Randomisation

After consent had been obtained participants were randomised into either the control group or intervention group. Randomisation was undertaken using a permuted-block approach. Randomisation blocks of two, four or eight participants were generated and randomly selected with the resultant allocation order placed into opaque sealed envelopes by an investigator not involved in recruitment or patient assessment (CW). The treatment for each participant was determined as per the random allocation sequence following completion of initial podiatric assessment.

All DRFUs (where a single participant had more than one ulcer) were numbered and documented according to anatomical location prior to randomisation. Only the treatment modality was randomised, therefore, when a single participant had more than one DRFU, all were treated using the same method.

Participants and treating podiatrists were unable to be blinded to treatment as neither method of debridement could be concealed. However, data analysis was undertaken without knowledge of the treatment allocation.

### Procedure

The DRFUs being treated in the intervention group were re-assessed after 6 weeks of treatment. If LFUD was no longer clinically indicated then this method of debridement was ceased and the ulcer was transitioned to the control treatment of NSSD. Clinical indications for ceasing LFUD included pain, small ulcer size or high levels of exudate.

As per the study criteria the ulcers included in this study were greater than 4 weeks old and therefore had received treatment prior to being enrolled in the study. The treatment prior to enrolment was determined at baseline through patient assessment and included surgical debridement, NSSD, autolytic debridement through dressings, topical negative pressure wound therapy, split skin grafting, offloading via podiatry felt padding, footwear or total contact casting.

### Statistical analysis

All analyses were undertaken using the intention to treat principle. The proportion of DRFUs that were healed by the 6 month follow up period was compared between the two treatment groups using Kaplan–Meier survival analysis approach. Due to the small sample size the planned logistic regression analysis was unable to be completed.

Pain and quality of life scores were not analysed statistically due to insufficient numbers of participants and as a result baseline comparability between the two groups could not be ensured.

## Results

A total of 10 participants with 14 ulcers were recruited to this study. Of the 14 ulcers, two ulcers (two different participants) were lost to follow up, one from each group. In one instance this was due to hospital admission to a different health service (intervention) and the other participant changed residential locations (control). Summative data for the primary outcome is presented in Table 2.

**Table 2:** Outcome data per ulcer

Control group			Intervention group		
Ulcers healed	Ulcers not healed	Lost to follow up	Ulcers healed	Ulcers not healed	Lost to follow up
5	0	1	5	2	1

**Fig. 1:**
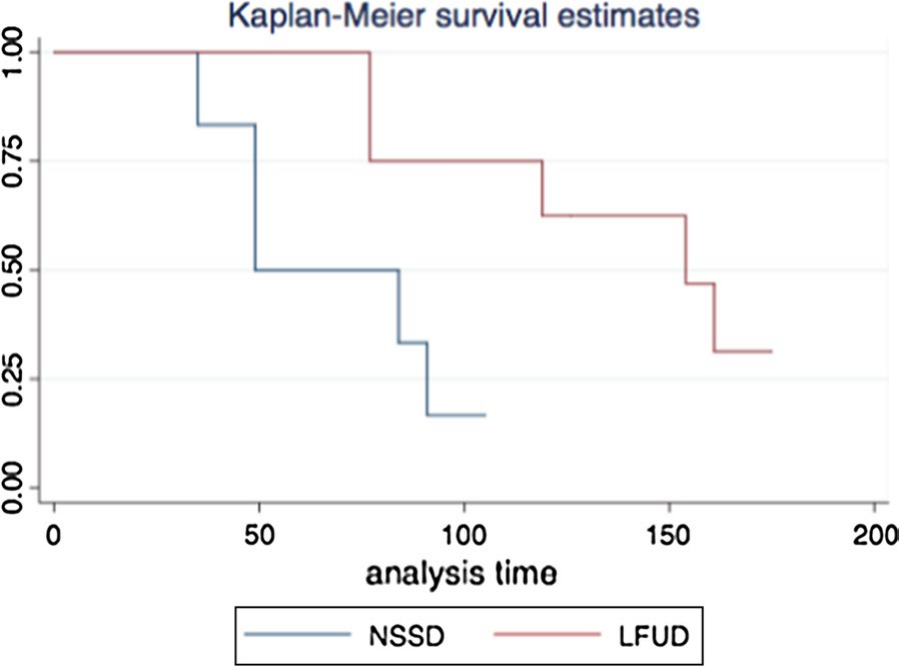
Kaplan–Meier survival estimates

A survival analysis estimating time to ulcer healing was undertaken and is presented in Fig. 1. Diabetes related foot ulcers treated with NSSD healed in a mean (SD) of 61.6 (24.4) days compared with those treated with LFUD healed in a mean (SD) of 117.6 (40.3) days.

The use of analgesia during treatment was comparable between both groups, with the same three ulcers from each group requiring some form of analgesia for every treatment. It was observed that pain levels increased during treatment but then returned to baseline levels after treatment.

The quality of life reported in both groups demonstrated an improving trend in scores as the ulcers progressed towards healing.

### Adverse events

During the follow up period 3 of the 14 ulcers were treated with oral antibiotics for minor soft tissue infections. No ulcer developed ascending cellulitis or osteomyelitis. No participants required surgical intervention, amputation or hospital admission during the follow up period. No other adverse events occurred throughout the study period.

## Discussion

Debridement is important to facilitate healing of DRFUs. This research investigated two methods of debridement available to clinicians that has not been widely studied. Whilst it was observed that ulcers treated with NSSD healed at a faster rate than those treated with LFUD, the sample size was too small to determine if this finding is significant. Despite the small sample size, our study findings are consistent with similar studies previously conducted comparing LFUD to NSSD in DRFUs. Four studies have previously been published, with three describing clinical trials involving randomisation and one using historical data as the control [17–20]. Although each of these studies concluded that DRFUs heal faster using NSSD compared to LFUD, between-study comparison was made difficult by heterogenic study design. These included differences between the type of LFUD performed, the frequency of treatments and variation in control treatments including wound dressings and offloading methods.

## Limitations

The greatest limitation of this study was the difficulty experienced recruiting participants. This makes it difficult to draw clinically significant conclusions. Furthermore, the planned statistical analyses, including health economics, were not undertaken.

Many attempts were made throughout the study period to address barriers to recruitment and increase participant numbers:

- Medical histories of all patients under podiatry care were reviewed by the primary investigator (LM) on a monthly basis to determine if study criteria were met and the patients could be considered for enrolment.
- Study criteria were pragmatically revised multiple times with approval from the relevant human research ethics committee.
- Recruitment was extended to include patients with DRFU attending Vascular Outpatient clinics.
- A second LFUD unit was secured on loan to allow a second podiatrist to potentially treat patients enrolled in the study at an additional site.
- Discussion ensued with the Podiatry Department at a second organisation with a view to implementing a multisite study.

Despite numerous attempts to increase recruitment rates the sample size fell short of numbers required to generate broadly applicable findings. These logistic problems were difficult to overcome and highlight the challenges of undertaking clinically unfunded research within populations with complex health needs.

There were a number of limitations that the research also encountered during the design and implications that future researchers should consider when undertaking this type of research with patients who have DRFUs:

- The type of ulcer dressings and pressure offloading used were not standardised.
- Inaccuracies in measuring ulcer depth where depth was less than 0.1 cm.

An important strength of this study design was the use of contact LFUD. Previously, only non-contact LFUD has been investigated in DRFUs [17–20]. Contact LFUD is thought to produce a cavitation effect, resulting in direct and immediate removal of nonviable tissue from the ulcer base. As the name suggests, noncontact LFUD produces the same phenomena but at a lower intensity and does not directly contact the ulcer surface. These slight variances mean that there is no debridement of necrotic tissue when noncontact LFUD is used [21]. This is also the first study to investigate the contact application of LFUD in DRFUs.

This study has revealed some interesting findings, which we believe would benefit from further investigation. Future randomised controlled trials would be of value to evaluate the clinical effectiveness of both debridement methods in the management of DRFUs. This patient population were found more likely to have multiple medical comorbidities that excluded them from ulcer debridement when subsequent patient lists were screened over the 2-year study period. This was an unexpected finding as the researchers designed this trial for patients with common traits applicable to DRFUs. Therefore, any future prospective research on this topic would benefit from consideration to a multisite study to ensure a large enough sample size could be reached. Additionally, the authors recommend including community care podiatry clinics where patients are more likely to be medically stable than those attending outpatient podiatry clinics based in the acute setting. Future research should also further investigate pain and quality of life assessment for patients between groups, as well as, the economic efficiency between both methods of debridement.

## Abbreviations

NSSD: non-surgical sharps debridement
LFUD: low frequency ultrasonic debridement
DRFU: diabetes-related foot ulcer
